# Elucidating the role of media nitrogen in augmenting the production of lignin-depolymerizing enzymes by white-rot fungi

**DOI:** 10.1128/spectrum.01419-23

**Published:** 2023-09-01

**Authors:** Vidya Pradeep Kumar, Manpal Sridhar, Samanta Ashis Kumar, Raghavendra Bhatta

**Affiliations:** 1 National Institute of Animal Nutrition and Physiology, Adugodi, Bangalore, Karnataka, India; Connecticut Agricultural Experiment Station, New Haven, Connecticut, USA

**Keywords:** depolymerization, laccases, peroxidases, nitrogen-limiting media, biomass, white-rot fungus

## Abstract

Indigenous white-rot fungal isolates *Schizophyllum commune*, *Phanerochaete chrysosporium*, *Ganoderma racenaceum*, and *Lentinus squarrosulus*, demonstrating the ability to depolymerize lignin of the crop residues, were studied for their potential to produce ligninolytic enzymes using modified production media under conditions of limiting and excess nitrogen for higher enzymatic expressions. Secretome-rich media on the investigation confirmed the successful production of lignin-depolymerizing enzymes, *viz*. laccase, lignin peroxidase, manganese peroxidase, and versatile peroxidase. Production of laccases and peroxidases was statistically significant in nitrogen-limiting media with and without the substrate, across all white-rot fungal cultures at 95% confidence interval. Nitrogen-limiting media with the substrate on analysis extracellularly expressed 99.27 U of laccase and 68.48 U of manganese peroxidase in *Schizophyllum commune*, while 195.14 U of lignin peroxidase was produced by *Phanerochaete chrysosporium. Lentinus squarrosulus* expressed 455.34 U of laccase and 357.13 U of versatile peroxidase with 250.09 U of laccase and 206.95 U of manganese peroxidase produced by *Ganoderma racenaceum* for every milliliter of the media used. Nitrogen-limiting media triggered the production of laccase during the initial stages of growth while the expression of peroxidases was predominant at a later stage. Also, this media evinced increased enzymatic yields with low biomass content compared to nitrogen-excess conditions. The extant study confirmed the positive influence of nitrogen-limiting media in the efficient production of ligninolytic enzymes and their suggestive degradation potential for environmental pollutants, making these enzymes a safe, clean alternative to the use of chemicals and the media to be effective for large-scale production of ligninolytic enzymes.

**IMPORTANCE:**

Lignin on account of its high abundance, complex polymeric structure, and biochemical properties is identified as a promising candidate in renewable energy and bioproduct manufacturing. However, depolymerization of lignin remains a major challenge in lignin utilization, entailing the employment of harsh treatments representing not only an environmental concern but also a waste of economic potential. Developing an alternative green technology to minimize this impact is imperative. Methods using enzymes to depolymerize lignin are the focus of recent studies. Current research work emphasized the efficient expression of the major lignin-depolymerizing enzymes: laccases, lignin peroxidases, manganese peroxidases, and versatile peroxidases from native isolates of white-rot fungus for several biotechnological applications as well as treatment of crop residues for use as ruminant feed in improving productivity. The importance of nitrogen in augmenting the expression of lignin-depolymerizing enzymes and providing a media recipe for the cost-effective production of ligninolytic enzymes is highlighted.

## INTRODUCTION

The molecular architecture of the lignin is a complex three-dimensional network held by an assortment of C-C and C-O ether bonds linking the phenylpropanoid units, making it highly recalcitrant for degradation. With crucial requirements of reduction of energy consumption and use of less toxic materials for effective lignin depolymerization, biological treatment has gained immense significance as an alternative green technology with minimal environmental impact. The demand for lignin-modifying enzymes is ever-increasing as they cater to the degradation and detoxification of environmental pollutants as well as tackling recalcitrant lignin, a constituent of wood and agricultural crop residues.

Microorganisms belonging to the wood rotting group are primarily lignin degraders and can potentially mineralize lignin accounting for about 25% of removable organic matter in the biosphere (1). Enzymes from bacteria and fungi are known to efficiently act on this complex polymer and initiate the process of delignification using oxidases and peroxidase enzymes. Both oxidases and peroxidases act on the lignin through redox reactions using molecular oxygen and hydrogen peroxide, respectively. The release of enzymes is highly regulated in nature and is unwavering when it comes to white-rot fungi (WRF) of the basidiomycetes group. Lignin-degrading enzymes generally contain lignin-modifying enzymes (LME) and lignin-degrading auxiliary enzymes that are necessary to complete the degradation process (2). LME includes phenol oxidase [laccases (LAC)] and heme-containing peroxidases (POD), namely lignin, manganese, and multifunctional (versatile) peroxidase along with the new enzyme heme-thiolate haloperoxidases (3), a versatile biocatalyst of the heme protein family. Auxillary enzymes like glyoxal oxidase, aryl alcohol oxidase, pyranose-2 oxidase, cellobiose dehydrogenase, and glucose oxidase (4) form part of the secretome of the WRF, supporting lignin degradation but cannot digest lignin on their own, in an aerobic aromatic ring cleavage mechanism (5).

The screening and documentation of WRF play a pivotal role as they can be exploited in various environmental and biotechnological applications (6, 7). It has been observed that WRF along with other saprophytic groups reduces lignin mineralization on nitrogen addition,(8) responding negatively to higher levels of nitrogen (9), and it is seen to appear in the advanced stages of wood degradation when microorganisms deplete the available nitrogen (10) in nature. Especially, the ligninolytic enzymes of WRF are increasingly gaining popularity in various sectors, and the presence of the carbon (C) and nitrogen (N) ratio as nutrients plays a pivotal role in triggering the reaction mechanism of lignin solubilization, contributing to the production of lignin-degrading enzymes by these groups. Soil mineral nitrogen is known to decrease lignin-degrading enzyme activity, especially in the late stages of litter decomposition (11, 12). Also, a reduction in decay rates of highly lignified plant litter was observed with an increase in nitrogen availability (13). Decomposers may increase their investment in lignin-degrading enzymes to acquire C from shielded cellulose (14) in nitrogen-depleted environments in the absence of high-energy carbon compounds and nitrogen limitation triggering large modifications in vegetative products and seed quality (15).

Limiting nutrients regulates the machinery responsible for the production of ligninolytic enzymes in WRF with special mention on the C and N ratios (16, 17). Right proportions of C and N in the medium influence the production of lignin-degrading enzymes, and the present study explored the potential of four indigenous cultures of white-rot fungi: *Schizophyllum commune* (*S. commune*), *Phanerochaete chrysosporium* (*P. chrysosporium*), *Ganoderma racenaceum* (*G. racenaceum*), and *Lentinus squarrosulus* (*L. squarrosulus*) to secrete ligninolytic enzymes. The cultures *S. commune* (18), *P. chrysosporium* (19), *G. racenaceum* (20), and *L. squarrosulus* (21) were considered to express enzymes, *viz*. laccase (LAC), lignin peroxidase (LiP), manganese peroxidase (MnP), and versatile peroxidase (VP), respectively, using modified liquid production media. Earlier studies relevant to lignin depolymerization recommend the use of the cell immobilization method for the continuous production of LAC from *S. commune* (22). Also, studies on the production of VP using *L. squarrosulus* (23) and MnP using *G. recenaceum* have been conducted to design a production medium using RSM (response surface methodology) with one factor at a time approach (24). Considering the media components from all the earlier studies (25, 26) and taking into account the importance of nitrogen limitation on the expression of ligninolytic enzymes in nature, the current study has given directions and attempts to (i) propose a media that enhances the production of lignin-depolymerizing enzymes across basidiomycetes WRF cultures, (ii) understand the relationship between growth and ligninolytic enzyme production, and (iii) establish the statistical significance of the liquid media used for enhancing the release of lignin-depolymerizing enzymes.

## MATERIALS AND METHODS

### Chemicals

All analytical grade chemicals were used in the present study. The substrates: 2,2′-azino-bis (3-ethylbenzthiazoline-6-sulfonic acid) (ABTS) and Reactive Black 5 (RB5) were procured from Sigma-Aldrich (USA). Azure B and manganese chloride (MnCl_2_) were purchased from Hi-Media.

### Organisms

Previously four WRF isolates were identified as potent ligninolytic enzyme producers and three of the native isolates were screened, identified, morphologically characterized, and deposited with Microbial Type Culture Collection (MTCC), Chandigarh. These cultures were procured from MTCC for revival, and the four WRF strains used in the present study are *S. commune* (MTCC 11893) (18), *P. chrysosporium* (MTCC 787), *L. squarrosulus* (MTCC 12922) (21), and *G. racenaceum* (MTCC 12928) (21). *Trametes versicolor* (MTCC 138) was used as a reference standard. The cultures were regularly subcultured on Malt Extract Agar (MEA) and stored at 4°C. All the cultures obtained from MTCC were lyophilized, and five rounds of subculturing in malt extract agar plates for 7–10 days at 30°C gave actively growing colonies. These were then considered for plate test assay.

### Plate test assay

Qualitative analysis for ligninolytic enzyme production was performed using 0.1% (wt/vol) guaiacol in potato dextrose agar (PDA) plates as substrate and 0.1% (wt/vol) ABTS in LAC detection agar (LDA) as the substrate to check for the presence of POD and LAC, respectively. Mycelial plugs (3 mm) of actively growing cultures were inoculated and incubated at 28±2°C for 5 days in an inverted position. LDA as previously reported (22) contained in grams per liter: KH_2_PO_4_ 1 g; C_2_H_8_N_2_O_4_ 0.5 g; MgSO_4_ 0.5 g; CaCl_2_ 0.01 g; Yeast Extract 0.01 g; CuSO_4_ 0.001 g; FeSO_4_ 0.001 g; MnSO_4_ 0.001 g; and 1.6% wt/vol agar) with 0.1% (wt/vol) ABTS. One milliliter of separately sterilized 20% wt/vol glucose solution was added to each 100 mL of autoclaved media. The plates were monitored daily for the development of green and brown colors for LAC and POD detection.

To confirm the presence of LAC and POD expression in plates, LAC detection liquid media and potato dextrose broth containing 0.1% (wt/vol) ABTS and 0.1% (wt/vol) guaiacol, respectively, were used as substrates. Twenty milliliters of the autoclaved media was poured into each 100-mL Erlenmeyer flask, inoculated with all four isolates (2 × 5-mm colony plugs), and incubated at stationary conditions for 7 days at 28°C±2°C. An assay for LAC was performed using 100 µL of the broth in 300 µL of 0.4M sodium acetate buffer (pH 5.2) and 0.5 µg/mL catalase. An assay for POD was performed using 100 µL of the broth in 300 µL of 0.4M sodium malonate buffer (pH 4.5) and 50 µL of 40 mM H_2_O_2_.

### Ligninolytic enzymes in liquid cultures

For liquid inoculations, actively growing cultures from MEA plates were peeled off using a 30-cm cell scraper and collected into a pre-sterile mortar with 5 mL of sterile distilled water. The mixture was blended in, using a sterile pestle to obtain a homogenized solution. From the solution, 100 µL of inoculum was dispensed into each tube (44 tubes × 4 tubes) (50 mL each) containing 10 mL of growth medium [media contained per liter: malt extract 30 g and peptone 5 g, supplemented with penicillin (12 mg/L) and streptomycin (2 mg/L) to arrest the growth of bacteria and incubated at 30°C for 3 days (80 rpm)]. After 3 days of incubation, growth media from each tube was decanted and replenished with 10 mL of production media. Four different media variations were used: nitrogen rich (NR), nitrogen limiting (NL), nitrogen rich with the substrate (NRS), and nitrogen limiting with the substrate (NLS). 0.016% wt/vol of the substrates were used in NLS and NRS media. The procedure was repeated independently for each WRF culture (44 × 4 tubes). An orbital shaker [30°C for 10 days (80 rpm)] was used to incubate the cultures continuously for 10 days (10 replicates were maintained), and four tubes (of each culture) were taken out every day after 24-hour incubation to assess for LAC and POD activity measurements.

### Composition of production medium

Liquid media (26) was modified for better ligninolytic enzyme production, based on the optimized methodology employed earlier. The modified liquid media contained per liter each of glucose 5 g, yeast extract 2.5 g, l-aspargine 2.5 g, and sodium nitrate 3 g. Potassium chloride 0.50 g, potassium dihydrogen phosphate 0.45 g, disodium hydrogen phosphate 0.17 g, magnesium sulfate heptahydrate 0.50 g, Veratryl alcohol 0.5 g, thiamine 0.0005 g, 10% Tween-80 solution 10 mL, oxalic acid 15 mM, copper sulfate 0.0005 g, manganese chloride 0.01 g, zinc sulfate 0.002 g, ferrous sulfate 0.005 g for NR broth, while all the constituents of NL media were the same except for l-aspargine (0.25 g), yeast extract 0.25 g, and sodium nitrate (0.30 g).

Substrate media: Each culture was grown in both NR and NL-modified media with and without their specific substrates. ABTS was used for *S. commune*, MnCl_2_ for *G. racenaceum*, Azure B for *P. chrysosporium*, and RB5 dye for the growth of *L. squarrosulus*. 0.016% wt/vol of all the dyes was prepared and 100 µL of the same was added to each flask containing 10 mL of the media. The total concentration of the substrate present in each flask was 0.005 µg. This makes the final concentration of the substrate in 10 mL to be 0.001 µM.

### Dry weight

Microbial biomass was deduced by filtering the media constituents through pre-weighed grade 4 quantitative filter papers (ashless) (12.5-cm diameter) followed by overnight drying at a constant temperature of 60°C. The weights of the filter paper post-oven drying were determined. The difference in weights constituted the biomass (grams).

### Enzyme assays

Cell-free culture filtrate was used as an enzyme source to determine the activities of ligninolytic enzymes. Cell-free supernatant of 500 µL was used to determine the presence of LAC and POD in general and LiP, MnP, and VP in particular. Enzyme assay for LAC was performed using 50 µL of ABTS (5 mM) in 300 µL of 0.4M sodium acetate buffer (pH 5.2) and 0.5 µg/mL catalase. About 100 µL of cell-free culture filtrate was added to the tubes to check for the presence of LAC. About 100 µL of distilled water in place of culture filtrate served as control. The reaction mixture was incubated for 10 minutes before taking the readings at A_420_ (ε_420_ = 36,000/M/cm) (M = Molar mass of the absorbing species). Enzyme activity was expressed in micromoles/minute/milliliter. One unit of LAC activity was defined as micromoles of ABTS oxidized per minute. Oxidation of ABTS by LAC alone was corrected by subtracting the activity in the presence of catalase from the activity in the absence of catalase.

The reaction mixture for guaiacol oxidation was performed using 300 µL buffer, 50 µL each of calcium chloride and guaiacol (5 mM), 10 µL hydrogen peroxide (H_2_O_2_) (40 mM), and 100 µL of cell-free culture filtrate. About 100 µL of distilled water in place of culture filtrate served as control. One unit of POD activity was defined as micromoles of guaiacol oxidized per minute. Reactions were initiated by the addition of 50 µL of 40 mM H_2_O_2_. The increase in absorbance was measured at A_470_ (ε_470_ = 26,600/M/cm). Enzyme activity was expressed in micromoles/minute/milliliter.

To determine the presence of LiP, 50 µL of 2 mM veratryl alcohol and 300 µL of 100 mM sodium tartrate buffer (pH 3.0) were used. About 100 µL of cell-free culture filtrate was added, and reactions were initiated by the addition of 50 µL of 40 mM H_2_O_2_. An increase in absorbance due to the oxidation of veratryl alcohol to veratraldehyde was measured at A_310_ (ε_310_ = 9,300/M/cm). LiP activity was also determined by the Azure B assay method where the reaction mixture contained 50 µL of 100 µM Azure B, 300 µL of 100 mM sodium tartrate (pH 4.5), and 50 µL of 40 mM H_2_O_2_. Oxidation of Azure B was determined by a decrease in absorbance at 651 nm.

The assay mixture for MnP quantification consisted of 50 µL of 1 mM MnSO_4_ and 300 µL of 100 mM sodium malonate buffer (pH 4.5). About 100 µL of cell-free culture filtrate was added. Reactions were initiated by the addition of 50 µL of 40 mM H_2_O_2_. An increase in absorbance for the oxidation of MnSO_4_ was measured at A_270_ (ε_270_ = 8,000/M/cm).

Both manganese oxidation and RB5 decolorization assays were used to determine the presence of VP. Oxidation of RB5 was determined in 300 µL of 100 mM sodium tartrate buffer (pH 3) with 50 µL of 10 µM RB5. The reaction was initiated by the addition of 50 µL of 40 mM H_2_O_2_, and the decrease in absorbance of RB5 was measured at A_598_ (24,000/M/cm). Oxidation of MnSO_4_ was measured by the increase in OD at 270 nm. All the assay reactions were carried out at room temperature.

### Statistical analysis

Statistical analysis was performed using OriginPro (version: 2022b) (27) software. R statistical software (version: 2022.7.2.576) (28) was used to generate the distribution plot of biomass across 10 days using qplot (lattice package). The influence of nitrogen concentration on growth and enzyme production levels was studied using the ggplot2 package along with the geom_smooth() function with source fungi and media as aesthetics, geom point with size parameter for biomass, color blue and position jitter as aesthetics and themes of R. Boxplot was also created to compare the impact of biomass on LAC activity and POD activity levels. A plot with a significant difference mark for pairwise comparison was generated using OriginPro 2022b. Probability distribution analysis was performed to evaluate the difference between biomass levels across WRF cultures when different culture media with limiting and excess nitrogen conditions were used and to observe for variations in LAC and POD production across cultures for different nutritional nitrogen conditions using the complete data set. Where the hypothesis for normal distribution was satisfied, parametric test analysis of variance (ANOVA) with Levene test for equal variance and a paired sample *t*-test were performed for biomass, to compare means between all the WRF isolates across the production methods used. Where the data set rejected normality, a comparison of inherent enzyme activities for LAC and POD across different media conditions for all four isolates of WRF was performed using non-parametric tests such as the Kruskal-Wallis ANOVA along with Mood’s median test, and Wilcoxon signed-rank test. To evaluate the differences between the production of LAC and POD enzymes by four different WRF isolates when different nutrient nitrogen conditions were used, Tukey *post hoc* and Dunn’s tests (Sidak correction) were performed for multiple comparisons both for parametric and non-parametric data, respectively. All the analyses set the confidence level to 0.05 to consider differences across different nutrient nitrogen media in all four cultures of WRF. All experiments were performed in triplicates.

## RESULTS

**Fig 1:**
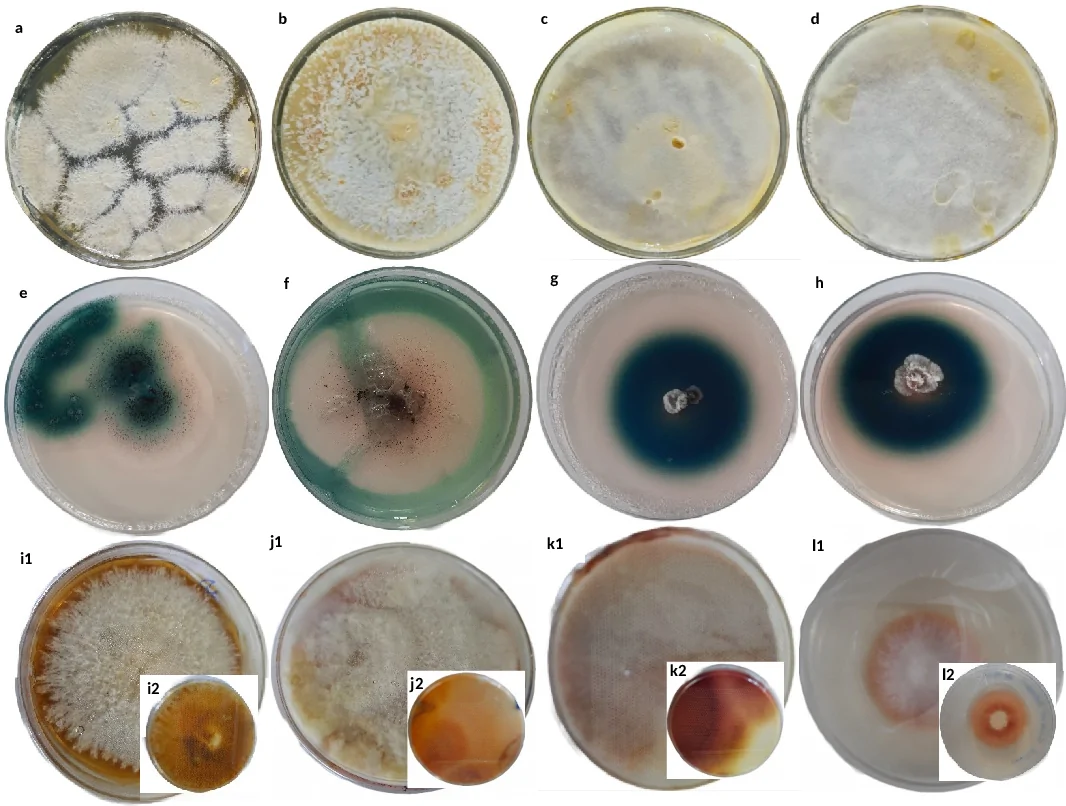
Screening of native white-rot fungal (WRF) isolates for ligninolytic enzymes. (**a**–d) Pure cultures on Malt Extract Agar (MEA) plates. (**e**–h) Plate assay for the oxidation of ABTS in the presence of LAC, which turns colorless medium to green. All four isolates in LDA plates were observed for the development of color, after 5 days of incubation at 30°C. The appearance of green color was seen in “e,” “g,” and “h” plates, while plate “f” showed the formation of green color only after flushing with 30% H_2_O_2_, indicating the presence of POD enzyme. (**i**–l) Plate assay for the oxidation of guaiacol: top view (**i1–l1**) and bottom view (**i2–l2**). WRF cultures: *Schizophyllum commune* (**a, e, i1, i2**); *Phanerochaete chrysosporium* (**b, f, j1, j2**); *Lentinus squarrosulus* (**c, g, k1, k2**); and *Ganoderma racinaceum* (**d, h, l1, l2**).

Four native isolates of basidiomycetes fungi, known to be potent producers of lignin-depolymerizing enzymes, were used in the present study. All the isolates grew profusely on malt extract agar plates (Fig. 1a and d), with whitish matte growth, as a characteristic of WRF.

Initial screening of pure cultures for ligninolytic enzyme production was performed on PDA plates containing 0.1% (wt/vol) guaiacol to check for the expression of POD and on LDM plates with 0.1% (wt/vol) ABTS for LAC production. Plate test assay using substrates confirmed the production of lignin-modifying enzymes by all four isolates qualitatively. The presence of POD in liquid cultures was confirmed by the oxidation of guaiacol. All the cultures showed positive results for guaiacol oxidation (Fig. 1i through l) by the formation of a brown/deep reddish halo in the culture plate confirming the release of ligninolytic POD enzymes extracellularly. However, this alone does not suggest the presence of all ligninolytic enzymes. The formation of green color around the colonies (Fig. 1e through h) on LDM plates confirmed the expression of the LAC enzyme. However, *P. chrysosporium* turned the plate green only when the plate was flushed with 30% H_2_O_2_ (Fig. 1f) suggesting the presence of any other ligninolytic POD but not LAC. The right proportion of the C to N ratio decides the efficiency of degradation. To corroborate the study and to understand the influence of nitrogen concentrations on the growth of the fungus as well as its influence on the production of lignin-depolymerizing enzymes: LAC, LiP, MnP, and VP, respectively, modified liquid media supplemented with suitable C and N source was used. Four variations of liquid production media: (i) NR, (ii) NL, (iii) NRS, and (iv) NLS were prepared. Liquid media NLS and NRS contained substrates such as ABTS, MnCl_2_, RB5, and Azure B. While RB5 is an azo dye, Azure B is an organic chloride and these are common dyes generally used in textile, fiber, cosmetics, paint, printing, leather industries, etc., and degradation of these dyes is an environmental concern. The potential isolates were initially grown in MEB for obtaining actively growing colonies. The pure active cultures grown in MEB were inoculated into each of the designed production media and incubated for 10 days under shaking conditions (80 rpm) at 30°C. Through the 10 days, biomass and ligninolytic enzyme production were monitored every 24 hours.

**Fig 2:**
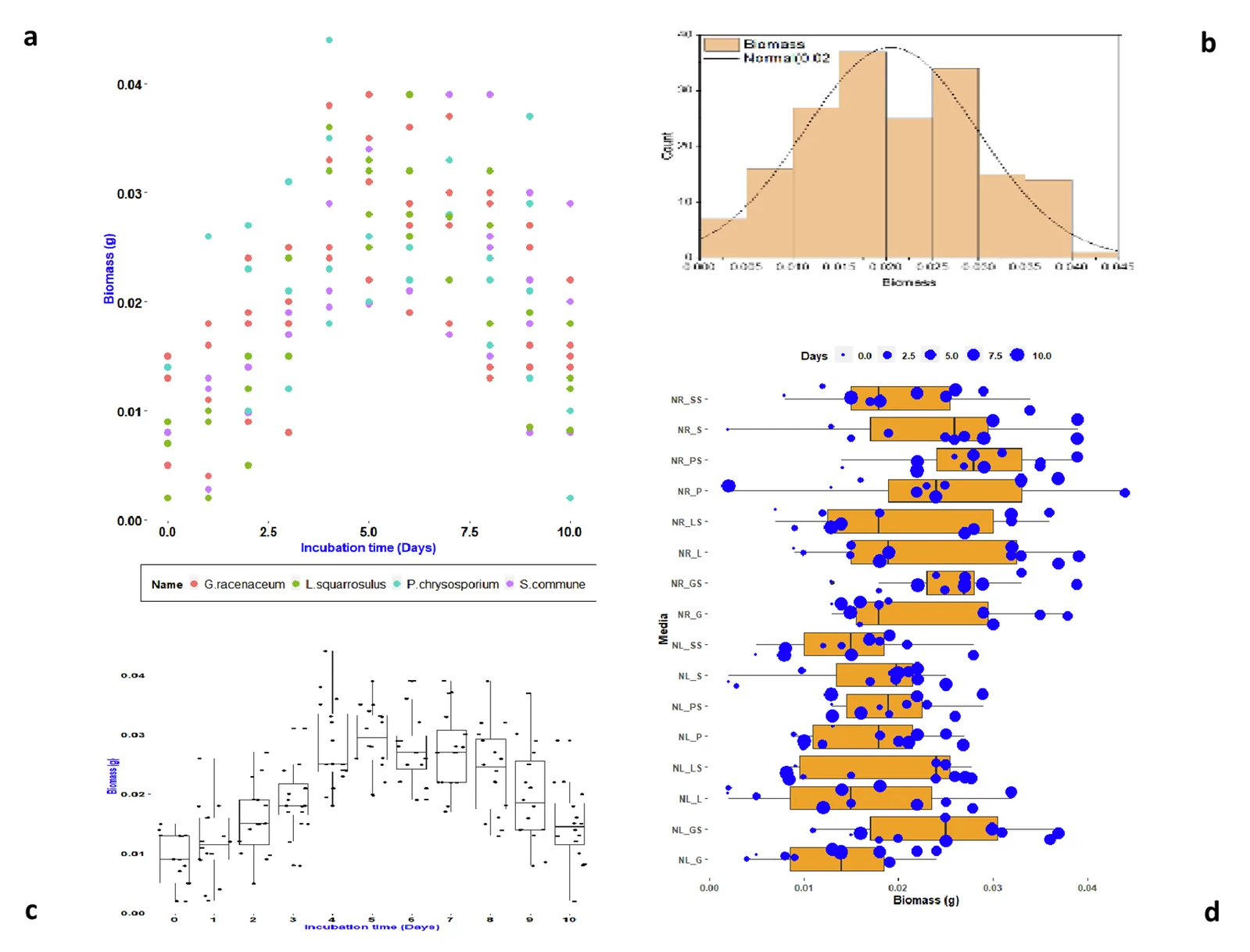
Distribution of biomass. (a) The time series graph summarizes biomass distribution through 10 days of incubation across four cultures of basidiomycete white-rot fungal isolates, under conditions of differing nitrogen in the production medium. The qplot () function in the ggplot2 package of R was used to plot the graph with geom_point () aesthetics “color,” mapping each species. Also shown is (b) the distribution plot and (c) the jitter boxplot to visually assess the distribution of the sample data. The box ranges from Q1 (the first quartile) to Q3 (the third quartile) of the distribution, and the range represents the IQR (interquartile range). The median is indicated by a line across the box. The “whiskers” on box plots extend from Q1 and Q3 to the most extreme data points. The schematic representation of the quartiles (
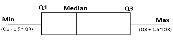
) (d) shows a ggplot with “Media” (*y*-axis) and Biomass (*x*-axis) across all the four cultures of WRF, taking setting “the number of incubation days” as the size parameter (blue). *Y*-axis description: nitrogen limiting (NL), nitrogen rich (NR), *S. commune* (**S**), *S. commune* with the substrate (SS), *P. chrysosporium* (P), *P. chrysosporium* with the substrate (PS), *L. squarrosulus* (**L**), *L. squarrosulus* with the substrate (LS), *G. racenaceum* (**G**), and *G. racenaceum* with the substrate (GS).

The plot (Fig. 2a) shows the distribution of biomass across 10 days of incubation. The biomass for *P. chrysosporium* in NL media showed an increasing trend from the 4th day with maximum biomass recorded on the 7th and 8th days, followed by *G. racenaceum*, with maximum development on the 7th day. *S. commune* later monitored the highest growth between the 6th and 7th day, and finally *L. squarrosulus* showed maximum growth on the 7th day. Except for *P. chrysosporium,* all cultures showed increased growth on the 7th day, irrespective of the production media used, declining slowly on further incubation because of nutrient depletion. The influence of the growth rate of the fungus across all fungal isolates using the number of days of incubation as the “size” parameter is depicted in Fig. 2d. It is very clear from the plot that NR media influenced the growth rates of the fungal isolates more prominently than the NL media, with *P. chrysosporium* showing maximum growth while *S. commune*, *L. squarrosulus*, and *G. racenaceum* displayed similar biomass levels in the NR medium. Distribution test for biomass using column descriptive statistics of origin pro-2022b at 0.05 confidence level showed that the data were significantly drawn from a normally distributed population (Fig. 2b), with the jitter boxplot (Fig. 2c), to visually assess the distribution across all media levels for the four fungal isolates studied. Since it fell within the framework of normal distribution, one-way ANOVA was performed with “incubation days” as a factor. The data showed the population means to be significantly different at 0.05 confidence level [*F* = 19.53, degrees of freedom (df) = 10, *P* = 2.03 × 10^−23^] with a data mean of 0.02. Hypothesis testing using one sample *t*-test for biomass across all media used for all WRF cultures gave a *t*-statistic of 29.33178 with mean ± SD of 0.02 ± 0.009 by 175 df showing a significant difference in the population mean from the test mean at 0.05 confidence level. Two sample *t*-test was conducted with biomass as the factor within NL, NR, NLS, and NRS media. It showed Mean_1_ − Mean_2_ to be significantly different from zero with a *t*-statistic of −2.0912 and df = 86 (*P* = 0.039). However, the two population variances were not significantly different at 0.05 onfidence interval (CI).

**Fig 3:**
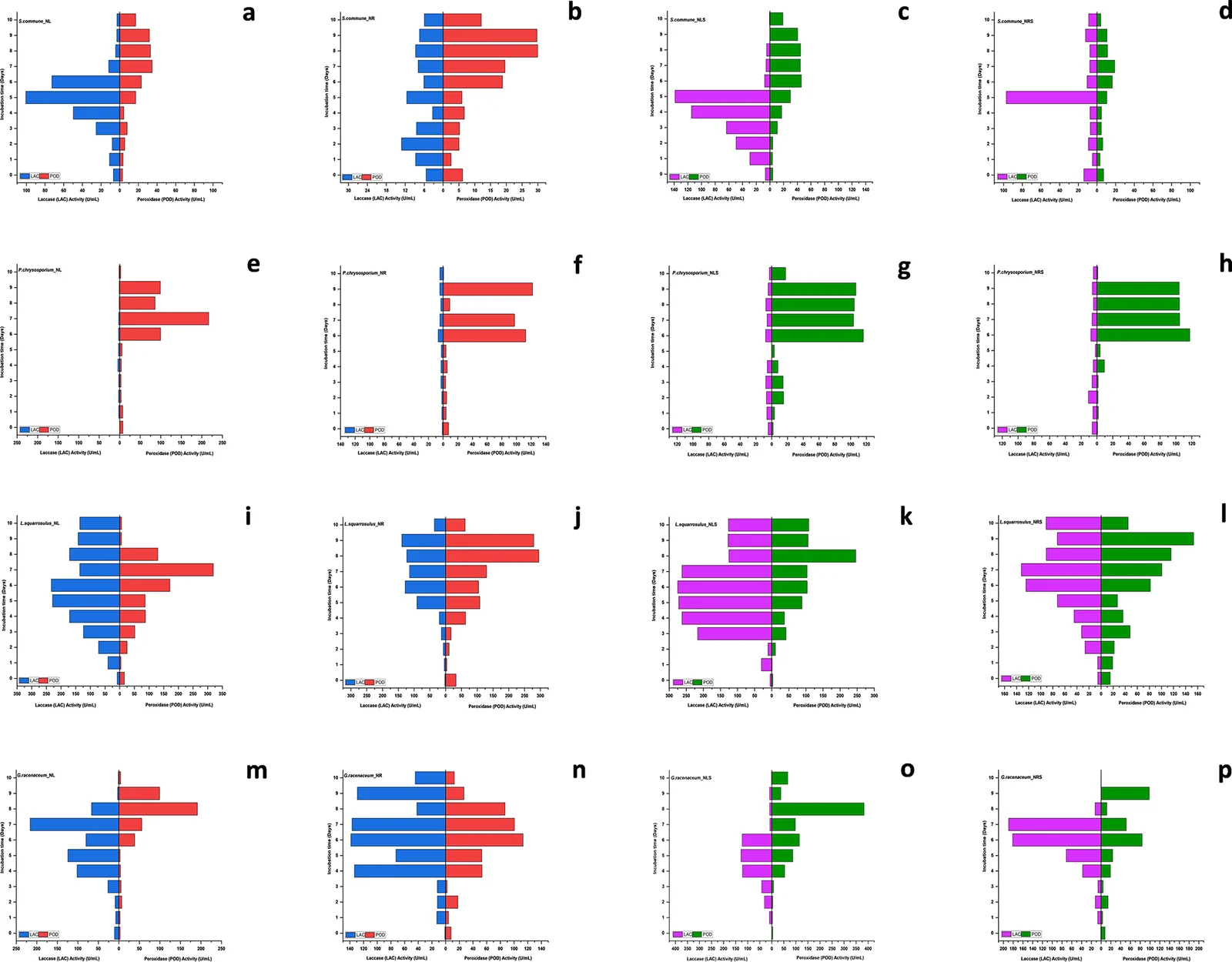
Distribution pattern across WRF isolates for ligninolytic enzymes. (a) Population pyramid statistical plots generated using OriginPro (2022b) represents the distribution of laccase (units per milliliter) and peroxidase (units per milliliter) production across four different cultures under various media conditions through 10 days of incubation at 28 ± 2°C. Each graph is divided down the center between laccase and peroxidase production levels with increasing activities growing from the center toward outside along the horizontal axis and incubation time (days) depicted on the vertical axis: *S. commune* (**a–d**); *P. chrysosporium* (**e–h**); *L. squarrosulus* (**i–l**); and *G. racenaceum* (**m–p**) “a,” “e,” “I,” “m” represents nitrogen-limiting media (NL); “b,” “f,” “j,” “n” represents nitrogen-rich media (NR); “c,” “g,” “k,” “o” represents nitrogen-limiting media with the substrate (NLS), while “d,” “h,” “l,” “p” represents nitrogen-rich media with the substrate (NRS). The plots have been generated using Origin Pro 2022b. One unit (1 U) of enzyme activity is defined as micromoles of product formed per minute.

Further, production media was monitored for the expression of ligninolytic enzymes. Fig. 3a through p shows population pyramid plots displaying shifts in the distribution of LAC and POD production levels for all four cultures across different media. Production of LAC in NL media for *S. commune* and *L. squarrosulus* was maximum on the 5th day, while *G. racenaceum* showed the highest production on day 7. Expression of LAC in NLS media for *S. commune*, *L. squarrosulus*, and *G. racenaceum* was more through the 4th–6th days, with a maximum on day 5. The quantity of laccase produced in NR and NRS media for all three cultures *S. commune*, *L. squarrosulus*, and *G. racenaceum* was consistently low when compared to NL media, but seemed to be stable throughout with the maximum production pushed to 6th and 7th days for *L.squrrosulus* and *G. racenaceum. S. commune* showed maximum LAC production on the 5th day in all the various media conditions used. *P. chrysosporium* did not express significant amounts of LAC across all the media tested. Production of POD was maximum on the 7th day in NL media for *S. commune*, *P. chrysosporium*, and *L. squarrosulus*, while *G. racenaceum* showed maximum expression of POD on the 8th day. All the cultures showed greater POD production on the 8th day in NLS media. Like NR and NRS media for LAC production, a similar trend was observed for POD production in NR and NRS media, with lower production rates compared to NL and NLS with greater stability. Maximum POD production was observed on the 9th day in NRS media for *P. chrysosporium*, *L. squarrosulus*, and *G. racenaceum* with maximum POD expression observed on the 7th day for *S. commune*. These graphs display a pattern showing LAC expression first in the media and expression of POD later. To further draw a clear picture, reaction rates were compared as a function of product formation in the time course graph generated for biomass, LAC, and POD activities for all four cultures together across all the media conditions used and are represented in Fig. S1. The graph was generated by pooling all the data across cultures and across media to establish an overall pattern of the influence of growth on enzyme production levels. It shows variations in the cell growth patterns and the comprehensive influence of biomass on lignin-depolymerizing enzyme productions, across a wide range of data points under different media conditions. The multi-panel plot reveals that with increasing biomass, LAC activity also increases at the initial days of logarithmic growth, with maximum activity recorded during the 5th and 6th days of incubation. Later during the stationary growth phase of the WRF isolates, after 5 days of incubation, the POD level leaps to a maximum on the 8th day of incubation. The plot depicts LAC activity levels to be prominent during the initial days of growth and POD activity levels to take charge in the later days, making it extremely explicit that, POD are released when the C source in the medium becomes minimal, along with N depletion. The boxplot (Fig. S2) generated considering total LAC and POD produced across WRF isolates in all the different media conditions used shows higher medians for POD compared to LAC confirming significant differences in enzyme production levels. Enzyme assay reactions for LAC using ABTS and assay for POD using guaiacol are depicted in Fig. S3. If the LAC enzyme is present in the medium, it converts ABTS to ABTS^+^ radical producing green color because of the formation of ABTS azine (420 nm). If the POD enzyme is present, the peroxide is quickly converted to water and oxygen, and the oxygen reacts with the guaiacol to produce a brown product, oxidized guaiacol (tetra guaiacol) at 470 nm. Further, the presence of each POD enzyme (LiP, MnP, and VP) or more than one in each production media was assayed and the activities were recorded. Under the production conditions used with modified production media, *S. commune* expressed LAC and some amounts of MnP, while *P. chrysosporium* produced LiP with very negligible amounts of LAC, *G. racenaceum* efficiently produced MnP along with LAC, and *L. squarrosulus* expressed VP into the medium with good amounts of LAC as well.

Distribution tests for LAC and POD production over 10 days of incubation rejected normal distribution. When the normality test was conducted for LAC activity across cultures, at 0.05 level, *P. chrysosporium* showed normal distribution to be significant (*P* ≥ 0.96); however, *S.commume*, *L. squarrosulus*, and *G. racenaceum* did not show a significant normal distribution of the population with very small *P* values at 0.05 confidence level. When POD activity across cultures was tested for normality, all the cultures rejected normality at 0.05 level (normality test results are available as part of the Supplementary data).

As the data set violated the assumption of a normal distribution when LAC and POD activities were concerned, a non-parametric Kruskal-Wallis ANOVA (KWANOVA) test was performed to statistically establish the relationship for enzyme production levels over 10 days of incubation. This test is an alternative to one-way ANOVA and compares the median among *k* populations as they do not require the assumption of normality which is used in situations when the data are categorical. KWANOVA for LAC across four cultures showed populations to be significantly different at 0.05 confidence level (chi-square = 72.78, df = 3, *P* = 1.08 × 10^15^) rejecting the null hypothesis that the samples came from the same population. The median test (median = 8.82) with *N* = 44 also showed a significant difference in populations at 95% CI rejecting the null hypothesis (*P* = 7.9 × 10^−15^). The test for LAC activity across media showed a significant difference in activity levels (chi-square = 84.51, df = 15, *P* = 1.03 × 10^−11^) with a maximum mean rank of 148.5 for *L. squarrosulus* in NL media followed by the same culture for NLS media with 143.18 ranks for *G. racenaceum*, then *S. commune* and *P. chrysosporium* in descending order. NR media also gave considerable activities; however, NL media in comparison stood as an outperformer for the best production media, across all the cultures used.

Further, the presence of each POD enzyme (LiP, MnP, and VP) or more than one in each production media was assayed and the activities were recorded. KWANOVA for POD across four cultures showed populations to be significantly different at 0.05 confidence level (chi-square = 21.44. df = 3, *P* = 8.48 × 10^−5^). The median test (median = 17.44) with *N* = 44 also showed a significant difference in populations at 95% CI rejecting the null hypothesis (*P* = 4.7 × 10^−4^). Among the four groups, the mean ranking of *L. squarrosulus* was more (129.46) with *G. racenaceum* falling next in line with a mean rank of 102.05. *S. commune* with a mean rank of 82.97 and *P. chrysosporium* with 39.5. The same test when performed for POD activity across different groups gave a chi-squared value of 21.45 showing a significant difference in populations at 0.05 level. The test for POD activity levels across different culture media used for all four WRF cultures also showed a significant difference in activity levels at 95% CI (chi-square = 27.33, df = 15, *P* = 0.026), with LAC activity across cultures showing the real significant difference with larger chi-square value of 72.78 in comparison to POD activity levels (chi-square = 21.44). POD activity levels across different media used showed a maximum ranking of 124 for NR_L followed by 122 for both NLS and NRS media for *L. squarrosulus*, with NL_GS, NR_GS, NLSS, NRSS, NL_PS, and NR_PS in descending order followed by NL media. The analysis directs that for improved POD activity levels both NLS and NRS were equally good compared to NL media alone and hence both NLS and NRS media could be considered for POD production by all the four cultures of WRF. Though the mean ranks obtained between NL and NR for POD activity were close, NL media still outperformed the NR media.

Significant differences in populations were observed at 0.05 level for WRF cultures, across different media conditions through 10 days of incubation, for *k*-sample non-parametric analysis of LAC (chi-square = 28.91, df = 10, *P* = 0.001). The populations were also significantly different for POD activity levels in all cultures through 10 days of incubation at 95% CI (chi-square = 92.68, df = 10, *P* = 1.57 × 10^−15^). Mood’s median test across WRF cultures for LAC showed a significant difference in activities at 95% CI for all the different variations of the media used (median = 8.82, chi-square = 77.09, df = 15, *P* > chi-square = 2.37 × 10^−10^). Nitrogen-limiting condition of *L. squarrosulus* culture displayed greater significance for the Mood’s median test with NL_L and NL_LS showing medians of 136.12 and 127.78, respectively, while *G. racenaceum* showed maximum significance for NL media with the substrate (NL_GS = 30.69). *S. commune* gave higher chi-square values under NL conditions than with NL with the substrate showing NLS of 10.83 (chi-square = 21.45) followed by NLSS of 9.37 (chi-square = 19.27). Considering NR and NRS media for these cultures, the chi-square values and medians were lesser than that of their NL and NL with substrate counterparts. The analysis clearly showed that NL media was better than NR for inducing ligninolytic enzyme production. Mood’s median test for POD also established a similar trend with a significant difference across WRF cultures when different media conditions were used (median = 17.44, chi-square = 26.90, df = 15, *P* > chi-square = 0.02) at 95% CI. The median for NL-LS was maximum at 88.38 and NL_GS at 53.49 and NL_L at 51.38. Then came *S. commune* with 18.98 and 17.66 for NLSS and NLS, respectively, followed by *P. chrysosporium* (7.09). The data for POD also showed that NL media conditions were more conducive to ligninolytic enzyme production than NR conditions. Most of the data points, depicting LAC activity in units per milliliter in comparison to biomass were below 0.02 g, and data points depicting POD activity in units per milliliter were above 0.02 g for both NL and NR media.

**Fig 4:**
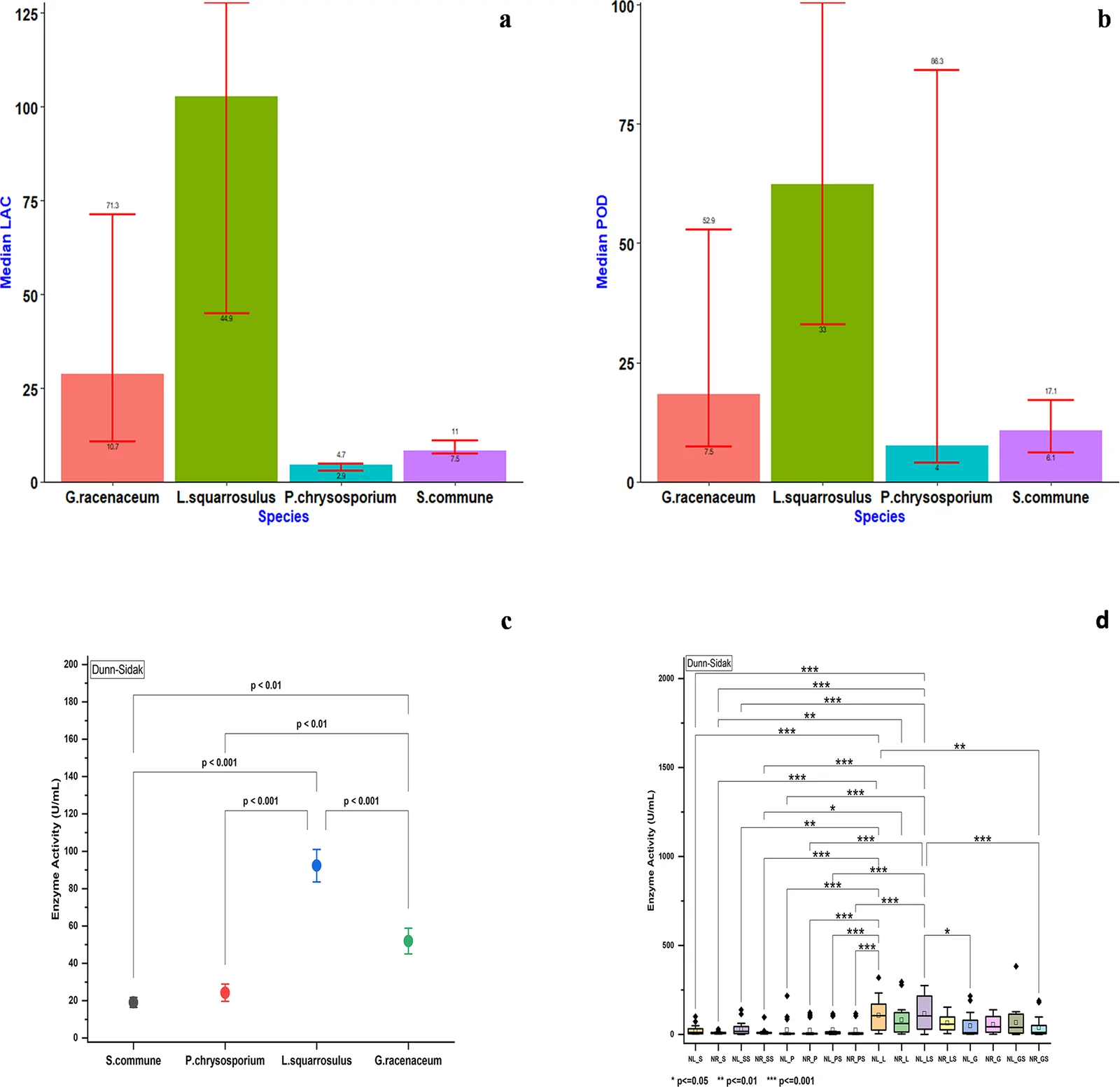
Medians with error bars for four WRF isolates producing ligninolytic enzymes. (**a–b**) (a) represents median values for laccase production across cultures and **(b**) denotes median values for peroxidase production across cultures, both at 95% CI. The red lines denote the SE of the median, and the columns denote the data median. **(c**) Pairwise comparison plot using the Dunn’s Sidak mean comparison method. A scatter plot was generated across four different WRF isolates for enzyme activity, excluding non significant values in groups with a significant mark generated by *P*-value with brackets. **(d**) A box chart with an asterisk denoting significant differences across all the different production media used for enzyme activity means across cultures. Both graphs are generated using Paired Comparison Plot App of Origin Pro 2022b software, at a 0.05 significant level and error bar in SE. One unit (1 U) of enzyme activity is defined as micromoles of product formed per minute.

When one sample non-parametric Wilcoxon signed-rank test, for total LAC activity levels, in different production media across four WRF cultures was performed, it gave a *Z*-value of 3.49 (*W* = 136), showing a significant difference in LAC activity levels of various production media across cultures (*P* = 4.82 × 10^−4^). Comparison of all media conditions for peroxidase production also showed a significant difference in the activity levels across cultures with a *Z*-value of 3.37 (*W* = 120) rejecting the null hypothesis (*P* = 4.82 × 10^−4^). Fig. 4a and b display the medians across four WRF isolates for LAC and POD, respectively. The Dunn’s test (Sidak-corrected *P* values), an alternate to Tukey’s test for non-parametric data, was performed to test for differences between all four WRF isolates for LAC and POD production levels among the different media conditions used. As a *post hoc* test in the ANOVA, it is used for multiple comparisons between groups.

Dunn’s *post hoc* test showed significant differences between groups (Fig. 4c): *L. squarrosulus* and *S. commune* (*P* < 0.001), *L. squarrosulus* and *P. chrysosporium* (*P* < 0.001), *G. racenaceum* and *S. commune* (*P* < 0.01), *G. racenaceum* and *P. chrysosporium* (*P* < 0.01), and *G. racenaceum* and *L. squarrosulus* (*P* < 0.001) with *L. squarrosulus* overlapping with three other WRF cultures showing the highly significant trend in enzyme production levels, while *G. racenaceum* with *S. commune* and *P. chrysosporium* showed marginally little less significance compared to *L. squarrosulus*, in terms of enzyme production levels.

The data across cultures for Dunn’s Sidak correction gave a positive trend establishing significant differences in enzyme production levels across all cultures of WRF strains. Significant differences between means for LAC and POD production levels across all the media used were also established using Dunn’s *post hoc* method with Sidak adjustment for family-wise error rate correction. The mean ± SE values for paired comparison using Dunn’s test for *S. commune* (12.71522 ± 1.96237), *P. chrysosporium* (16.19049 ± 3.25489), *L. squarrosulus* (61.55789 ± 6.93046), and *G. racenaceum* (34.63158 ± 5.06493) demonstrated that *L. squarrosulus* mean value to be higher than all other three WRF cultures for various media used. Multiple comparisons using Dunn’s Sidak test across WRF cultures (Fig. 4d) for enzyme production showed significant differences in NL and NLS media for *L. squarrosulus*, *S. commune*, and *P. chrysosporium.* Also, significant differences were observed in NR and NRS media for *S. commune* and *P. chrysosporium*. A similar trend was seen in NLS and NR of *S. commune* with all media variants of *P. chrysosporium* (NL, NR, NLS, and NRS) and finally NRS of *G. racenaceum* with NLS of *L. squarrosulus* (*P* ≤ 0.001). Also, a substantial difference was observed between NL, NR, NLS, and NRS of *L. squarrosulus* and *S. commune* and with NRS of *G. racenaceum* (*P* ≤ 0.01).

**Fig 5:**
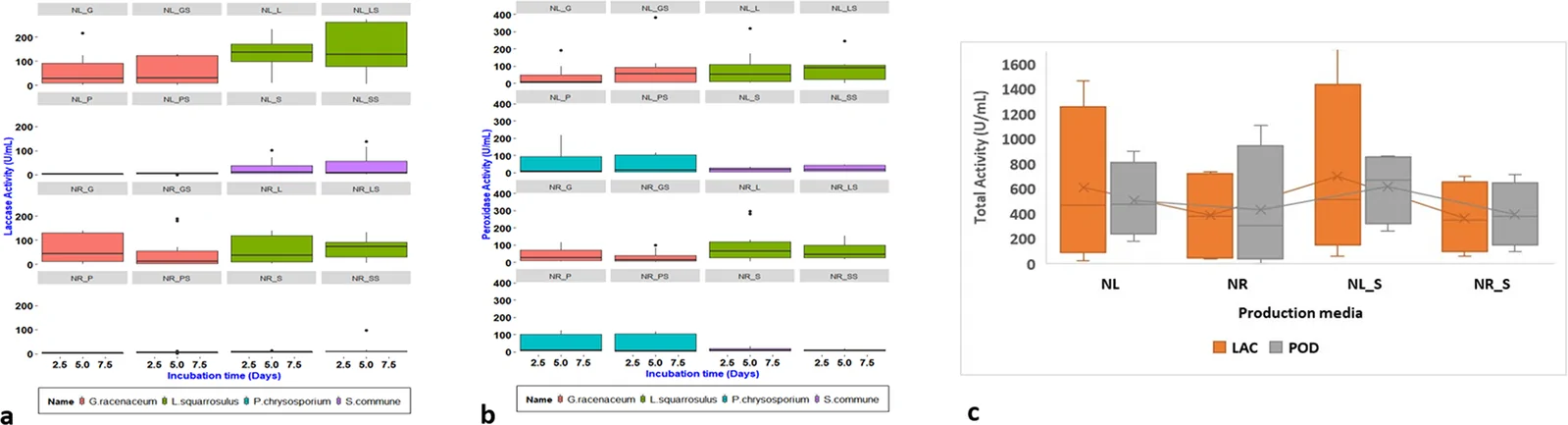
Trellis plot, a grouped boxplot with ggplot2 (a and b). The graph is partitioned into multiple channels by levels of the different production media used across cultures through 10 days of incubation. The facet grid () function was used to arrange the 16 panels into four rows and four columns. The panel plots are generated using incubation time (days) on the *x*-axis with production media being the facet and (a) laccase activities and (b) peroxidase activities in units per millliliter on the *y*-axis. (c) The box and whisker chart represents the schematic distribution of data for total enzyme activities of both laccases and peroxidases, in production media across four different WRF cultures. The boxes and the whiskers denote the production of the ligninolytic enzymes under limiting nitrogen (NL) and nitrogen-rich (NR) conditions for all the isolates, with lines connecting the means of laccase and peroxidase activities simultaneously for each production media used. “S” in the media denotes the substrate. Horizontal lines within each box represent the median. One unit (1 U) of enzyme activity is defined as micromoles of product formed per minute.

A grouped boxplot generated using the ggplot2 package of R, gave a clear demarcation for all the cultures on the production of LAC and POD across media for 10 days of incubation. It is very clear from the Trellis plot (Fig. 5a) that increased levels of LAC production were seen in NL_LS followed by NL_GS and NLSS for *L. squarrosulus*, *G. racenaceum*, and *S. commune* in NL media with substrates compared to NL media. From the data obtained, it is very evident that each species outperformed in enzyme production levels in NL media compared to NR media. A similar pattern of enzyme production was evidenced (Fig. 5b) for POD as well across incubation days, with NL_LS and NL_L of *L. squarrosulus* taking the lead with *G. racenaceum*, *P. chrysosporium* falling next in line.

**Fig 6:**
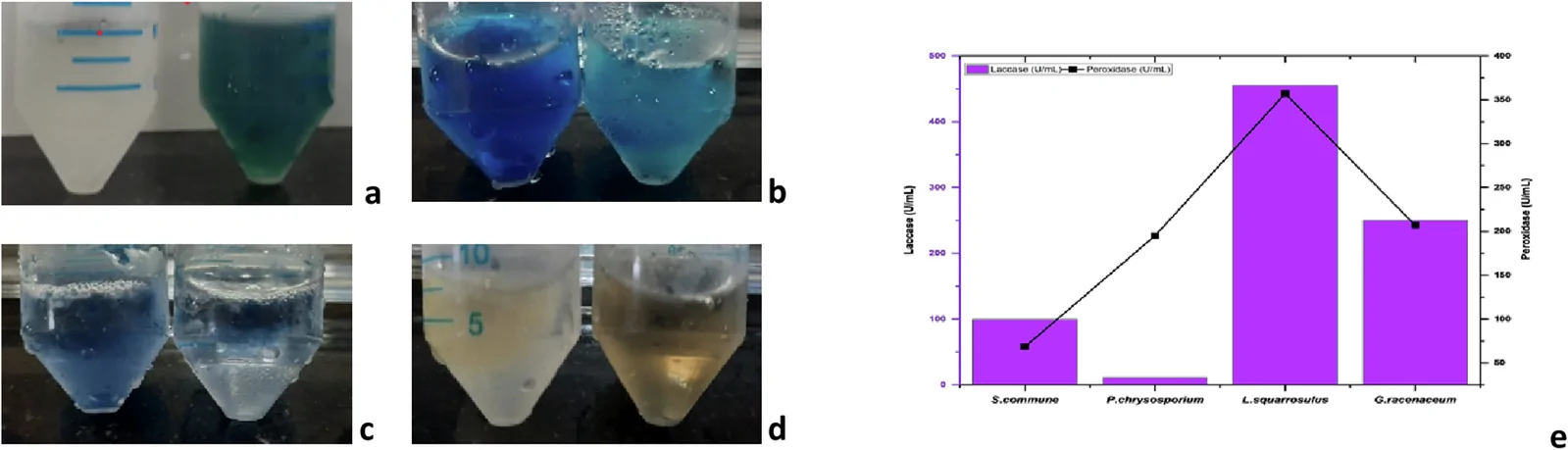
The secretome activity profiles of lignin-modifying enzymes (LMEs) across four isolates of WRF cultivated under submerged conditions for a period of 10 days. The photographs indicate a visual representation of the decoloration studies under limiting nitrogen conditions. The photographs of the first tubes of each picture (**a–d**) were captured on day 3. The pictures of the second tubes for each culture were captured on (a) *Schizophyllum commune* (day 5), (b) *Phanerochaete chrysosporium* (day 7), *(*c*) Lentinus squarrosulus* (day 9), and (d*) Ganoderma racinaceum* (day 6). (e) The 2Y plot was generated using OriginPro 2022b software and represents enzyme activities in units per milliliter for laccases and peroxidases. The *y*-axis values denote the mean of three replicates. One unit (1 U) of enzyme activity is defined as micromoles of product formed per minute.

Comparison of the total enzyme activities of LAC and POD production (Fig. 5c) for all four isolates together for NL, NR, NLS, and NRS media offered a remarkable confirmation directing the use of NLS in increasing the expression of ligninolytic enzymes extracellularly. Finally, the experiment was again repeated using 0.001 µM of the substrate in nitrogen-limiting and NR production media (10 mL each) and inoculating with mycelial disks (2 × 2 mm) of pure cultures into each tube. Assaying of the secretome activity profiles of all the four lignin-depolymerizing enzymes in all the four isolates of WRF, grown in nitrogen-limiting media using substrates ABTS, Azure B, MgCl_2_, and RB5 (Fig. 6) for the production of LAC, LiPs, MnPs, and VPs, respectively, showed decoloration of the dyes confirming the production of lignin-degrading enzymes. Fig. 6a showed green decoloration of the NLS media with *S. commune* containing the substrate ABTS, confirming the extracellular expression of the LAC enzyme. NL culture broth with Azure B (the major metabolite of methylene blue) showed significant degradation of this organic chloride (Fig. 6b) suggesting the production of LiP by *P. chrysosporium*. Decoloration of RB5 (Fig. 6c) in NL tubes by *L. squarrosulus* indicated the production of VP, and the appearance of brown pigmentation (Fig. 6d) in MnCl_2_ containing NL media confirmed the production of MnP by *G. racenaceum*. In comparison, among the four cultures, *L. squarrosulus* exhibited maximum stability for LAC. *S. commune* recorded maximum activity on the 5th day in NR medium and the same trend was observed for *G. racenaceum* as well, with maximum activity on the 5th and declining subsequently. *S. commune* exhibited a little amount of MnP activity in NL media on the 8th day. *P. chrysosporium* displayed maximum LiP activity on the 7th day in the NL medium, while the maximum activity of LiP was observed on the 9th day in the NR medium. *L. squarrosulus* displayed a higher activity of VP on the 8th day, increasing from the 6th day in NL medium while the same trend was observed for NR as well. NL and NRS showed maximum activity on the 8th and 9th days. Similar results were seen in MnP expression by *G. racenaceum* on the 8th day in the NL and NLS medium, showing significant production of MnP. The 2Y-axis plot (Fig. 6e) shows the amount of LAC and POD produced by each culture in NL culture tubes where changes in the color of the medium have been observed. *S. commune* produced both LAC (99.27 U/mL) and MnP (68.48 U/mL), while 195.14 U of LiP was produced by *P. chrysosporium*. A negligible amount of LAC (11 U/mL) was recorded for this species under the growth conditions used. *L. squarrosulus* expressed 455.34 U/mL of LAC and 357.13 U/mL of VP, while 250.09 U/mL of LAC and 206.95 U/mL of MnP were produced by *G. racenaceum*. The study clearly showed that NL media influenced the expression of lignin-depolymerizing enzymes across all the basidiomycetes isolates used.

## DISCUSSION

In natural habitats like wood, forest litter, etc., the basidiomycetes WRF act on the heteropolymer lignin by oxidative depolymerization process releasing ligninolytic enzymes as part of the secondary metabolic phase, under conditions of nitrogen limitation, during the mycelial growth stage. WRF produces more than one ligninolytic enzyme or an array of lignin-modifying enzymes to efficiently depolymerize lignin in the wood. Four native WRF isolates *S. commune*, *P. chrysosporium*, *L. squarrosulus,* and *G. racenaceum* considered in the present study were identified as efficient producers of ligninolytic enzymes LAC, LiP, VP, and MnP, respectively. Most WRF isolates are known to be LAC producers, and the plate test assay using ABTS turned the colorless agar medium to green due to the oxidation of ABTS to ABTS azine in the presence of LAC (18, 29). Substrate oxidation by LAC is a single-electron reaction generating a free radical. The initial product of the reaction is typically an unstable phenoxy radical that can be converted to quinone in a second reaction catalyzed by the enzyme (30). The isolates, *S. commune*, *G. racenaceum*, and *L. squarrosulus* showed evidence of the presence of extracellular enzyme by the formation of green color around the colonies, confirming LAC production. The formation of green color on the LDM plate with *P. chrysosporium* was formed only after the addition of 30% H_2_O_2_ advocating the production of the POD enzyme. This phenomenon is quite in agreement with the study of Hatakka (31) and Huang et al. (32), where, by using the WRF *P. chrysosporium*, the details of the lignin degradation mechanism have been elucidated by the production of multiple isoenzymes of lignin and MnP, which do not produce LAC. Plate test assay using guaiacol showed the formation of a reddish-brown halo around the colonies, confirming POD enzyme production. Guaiacol is a methoxylated phenolic (1-hydroxy-2-methoxybenzene) compound, the oxidized products of which are dark reddish-brown quinones and an insoluble polymerizate formed by random phenoxy radical reactions (33). Many studies have previously reported the oxidation of guaiacol by LAC (34, 35) and the oxidation of ABTS by POD (36, 37). To correct for guaiacol decoloration by LAC and ABTS decoloration by POD and to confirm plate test assay results for LAC and POD productions, an assay for LAC was performed using catalase correction; an assay for POD was performed using H_2_O_2_. All the pure cultures were then considered for liquid cultivations, where variations in the composition of the nitrogen source were used in the production medium. Filamentous fungi adapt to changing environmental conditions by altering their metabolism under nutrient-limiting conditions with C excess and N/P (phosphorus) limitation (38). Earlier studies by Yan and Wu (39) also support this statement, where degradation of polycyclic aromatic hydrocarbons by WRF was favored under limiting nitrogen conditions. All four isolates grew profusely in the modified production medium used with maximum growth recorded on the 7th and 8th days across different media conditions. The growth takes a logarithmic phase during the initial days of cultivation, because of the presence of media N, which is required for cell multiplication. Growth was more prominent in NR media than in NL media in comparison, as ample N source was available for growth in the former. It has also been earlier reported that microbial cell growth is enhanced in the presence of a nitrogen source (40). Similar results of highest growth were obtained by Lonardo (41) while growing *Trichoderma harzianum* and *Mucor hiemalis* when nitrogen levels were more. Kachlishvili et al. (17) also observed that additional N (20 mM) repressed MnP production by all fungi tested in his studies. Lashermes et al. (42) noticed an increased growth for a litter-decomposing fungus grown on maize litter, with increasing N availability in the plant material. This trend in growth pattern was observed because organic N sources such as yeast extract support rapid growth and high cell yields (43). A study of the time course graph across cultures in the production media for 10 days period clearly explained biomass accumulation to be maximum on the 7th day, thereby declining slowly onwards. It was observed that irrespective of the production media used, LAC expression was high during the initial days of growth while POD expression levels were maximum at a later stage. This trend was observed across cultures. A similar trend of stimulated biomass accumulation was also observed by Kachlishvili et al. (17) under nitrogen-sufficient conditions with sufficient quantities of LAC in the medium. This is in line with the results obtained in the study across cultures, emphasizing the fact that LAC levels increased after 3 days of growth with increasing biomass yields, reaching their maximum during the initial days of growth. Higher VP activities were observed in NL media with the enzyme reaching its maximum activity of 33 U/mL after 21 days of cultivation (25). The results obtained in the present study under similar conditions for VP with the modified production media recorded 357.13 U/mL with a 10-fold increase in activity. The cultures showed the production of POD effectively after the initial days of incubation, when the biomass growth kind of reached a plateau, indicating nutrient depletion conditions. A similar trend of lignin mineralization was observed by Kirk and Farrell (44) while using *P. chrysoporium*, on cessation of vegetative growth and onset of secondary metabolism, setting a prototype for WRF nutrient mineralization.

The use of organic compounds and azo dyes as substrates in NL media was designed to check if the extracellular enzymes secreted by the WRF strains could oxidize and break them down, as these dyes if inhaled and on prolonged skin contact causes respiratory as well as dermal irritation, respectively (45). ABTS, an organic compound, is also a respiratory and skin irritant and needs proper disposal. The non-phenolic dye ABTS is oxidized by LAC produced by *S. commune* to the more stable and preferred state of the cation radical. The concentration of the cation radical responsible for the intense blue-green color is correlated to enzyme activity (46). MnP activities were also recorded in this fungus. The presence of MnCl_2_ in the production medium along with NL conditions could be the reason behind the production of MnP by this WRF culture. An increase in the MnP levels using *Pleurotus ostreatus*, with yeast extract as the N source, was recorded in media with manganese, speculating the presence of manganese to affect the production of MnP (47). *S. commune* was also used by Horisawa et al. (48) and Kumar et al. (49) to study ligninolytic enzymes in solid-state fermentation and liquid fermentations to study depolymerization of agro-industrial residues, respectively. A very negligible amount of LAC was monitored in the media during the initial growth stages while *P. chrysosporium* was inoculated into the production medium. Maximum POD activity was monitored on the 7th day of growth, with activity decreasing gradually on the 8th day. Little LAC activity was monitored on the 7th and 8th days in NR media, however, consistently less than that of POD in the media used. Dittmer et al. (50) observed a minor peak at 600 nm of the presence of LAC activity in *P. chrysosporium*, confirming the presence of LAC isoforms under nutrient-sufficient conditions. Maximum POD activity was recorded on the 9th day, dependably less than that obtained in NL media. The culture broth with Azure B (the major metabolite of methylene blue) showed significant degradation of this organic chloride suggesting oxidation of the phenolic group at the carbon bearing the azo bond to produce a radical group (51). Haq and Raj (52) showed more than 90% decoloration of Azure-B dye using LiP enzyme-producing *Serratia liquefaciens* in a mineral salt medium (MSM) at 30°C. Also, one of the other reasons for the degradation of this environmental pollutant is the C source in the production medium. Studies by Saranraj et al. (53) and Kumaran et al. (54) have validated the decoloration of reactive textile dyes using *Bacillus thuringiensis*, *Bacillus subtilis*, *Bacillus cereus*, *Alcaligenes* sp., and *Nocardiopsis alba* with the addition of sucrose and degradation of Brilliant Black BN using Actinobacteria and a *Paraburkholderia* sp. with the addition of glucose, respectively, showcasing the influence of C source in dye degradation. When *G. racenaceum* was considered for ligninolytic enzyme production, both LAC and POD were expressed extracellularly. Measurement of POD activities in NL media, along with LAC, demonstrated the production of MnP as well, with the highest activity obtained using MnCl_2_ along with H_2_O_2_. Maximum activity was recorded on the 7th day in NL medium. This culture gave better results in both NLS and NRS media because MnCl_2_ as manganese influenced the production of MnP extracellularly. Rodrigo et al. (55) studied the effect of manganese on the secretion of MnP by the basidiomycete *Ceriporiopsis subvermispora*, confirming extracellular MnP secretion only when manganese is added, which otherwise would remain in the intracellular compartments. The presence of MnP in the media is validated by the presence of manganese (III) ions that could have been stabilized by the presence of FeSO_4_ in the media components giving brown/black manganese oxide (Mn_2_O_3_). Kuan and Tien (56) observed the stabilization of Mn^3+^ ions in the catalytic cycle of MnP because of oxalic acid, as a metal chelator, serving as a donor or acceptor of electrons (57). *L. squarrosulus* for VP production gave very interesting results and appeared to be taking the lead when it comes to producing ligninolytic enzymes. In comparison to NR, NL gave better results for this culture as well. The isolate along with *G. racenaceum* produced LAC enzyme, during the initial days of growth. High redox potential aromatic dye like RB5 is a substrate specific to VP. Decoloration of RB5 in NL media by *L. squarrosulus* confirmed the production of VP. Graz and Jarosz-Wilkołazka (25) also studied the secretion of VP in NL media by *Bjerkandera fumosa*, while Knop et al. (58) demonstrated that VP1 is responsible for RB5 oxidation and that effective decoloration proceeds at pH 3.5. The pH of the medium initially observed on day 1 in the production medium was around 7.0 and eventually decreased to 5.5 to 6.0 where maximum LAC levels were recorded. Eventually, the pH further reached around 3.8 for all isolates, in line with the above statement made, except for *S. commune* and *P. chrysosporium* where POD levels were monitored at pH 5.5 and 4.5, respectively. This also supports the observation that LAC activities were higher during the initial days of growth where pH was around 6.0 and declined slowly as the pH becomes more acidic and the LAC levels appeared to be more stable in NR and NRS media compared to NL and NLS. Barber-Zucker et al. (59) used VP constructed from sequences and confirmed decoloration of the dye RB5 by the oxidation of the high-redox potential surface-active tryptophan. In line with this, previously, Ravichandran et al. (23) observed the decoloration of RB5 and kraft lignin by immobilized *L. squarrosulus* in an optimized production medium, with Fourier-transform infrared spectroscopy (FT-IR) peaks confirming the cleavage of azo bond in RB5. Decolorization of organic substrates was monitored on the 5th, 7th, 6th, and 9th days of growth for *S. commune*, *P. chrysosporium*, *G. racenaceum*, and *L. squarrosulus*, respectively, releasing ligninolytic enzymes into the culture media. Many factors in the media trigger elevated enzyme expression extracellularly. Enzyme production levels are also enhanced because of the iron ions, which are solubilized and sequestered by siderophores produced by many fungal species in the early stages of wood decay (60). The enzyme active site is protected by the addition of 0.1% of detergents such as Tween 80 or non-reactive foam-stabilizing agent such as polyethylene glycol (PEG) (61) along with trace element solution that includes calcium, manganese, iron, zinc, copper, and magnesium and is necessary for the induction of the lignin-degrading POD system. All these components in the media synergistically influenced the elevated expression of these enzymes. A comparison of the complete data set suggested that NL media is more favorable for ligninolytic enzyme production than NR media.

The current work holds vital significance in highlighting the conditions for improved production of ligninolytic enzymes: LAC, LiPs, MnPs, and VPs from four native WRF isolates and their applications in organic dye decoloration. N availability in wood is limited as compared to soil organic nitrogen and the presence of optimum nitrogen triggers lignin-depolymerizing enzyme production. It is a well-established fact across basidiomycetes that during fruiting body formation, the demand for nitrogen utilization is high and thus the ligninolytic enzymes become poorly expressed during this stage of growth. However, through the asexual growth cycle of the fungus under submerged cultivation conditions, depolymerizing enzyme production is triggered. There is an inverse relationship between N demand and ligninolytic enzyme production, clearly demonstrated in the fact that during fruiting body formation, ligninolytic enzyme production is minimal. This becomes the impetus toward moving from a solid state to submerged fermentation methods for enhancing lignin-depolymerizing enzyme production under nitrogen-limiting conditions, more so as submerged fermentation supports easy regulation in terms of balancing media components. The impact of N concentration on oxidative ligninolytic enzyme production by four isolates of WRF was evaluated and results confirmed the utilization of nitrogen-limiting broth for the growth of WRF could positively influence the active expression of lignin-depolymerizing enzymes extracellularly. The right proportion of N in the production media, for the growth of WRF, offers considerable promise for the release of lignin-modifying enzymes effectively for use in several biotechnological applications. The encouraging results obtained were instrumental in the design of a nitrogen-limiting production media for large-scale expression of lignin-depolymerizing enzymes using a cost-effective, sustainable, and greener approach.

## SUPPLEMENTAL MATERIAL

Fig. S1 spectrum.01419-23-s0001.docx Time course plot of biomass, laccase, and peroxidase across different production methods.

Fig. S2 spectrum.01419-23-s0002.docx Mean, median, and variance between total laccase and peroxidase across different production methods.

Fig. S3 spectrum.01419-23-s0003.docx Assay for LAC and POD in liquid cultures.

ASM does not own the copyrights to Supplemental Material that may be linked to, or accessed through, an article. The authors have granted ASM a non-exclusive, world-wide license to publish the Supplemental Material files. Please contact the corresponding author directly for reuse.

## References

[1] Huang X-F , Santhanam N , Badri DV , Hunter WJ , Manter DK , Decker SR , Vivanco JM , Reardon KF . 2013. Isolation and characterization of Lignin-degrading bacteria from Rainforest soils. Biotechnol Bioeng 110:1616–1626. doi:10.1002/bit.24833 23297115

[2] Moreira C , da Silva J , Maciel GM , da Silva SM , Inácio FD , Bracht A , Peralta R . 2013. Involvement of Lignin-modifying enzymes in the degradation of herbicides. agricultural and biological sciences, p 165–187. In Andrew J , AK Jessica (ed), Herbicides - advances in research. Intech. doi:10.5772/51496

[3] Hofrichter M , Ullrich R . 2006. Heme-thiolate haloperoxidases: versatile biocatalysts with biotechnological and environmental significance. Appl Microbiol Biotechnol 71:276–288. doi:10.1007/s00253-006-0417-3 16628447

[4] Levasseur A , Drula E , Lombard V , Coutinho PM , Henrissat B . 2013. Expansion of the enzymatic repertoire of the CAZy database to integrate auxiliary redox enzymes. Biotechnol Biofuels 6:41. doi:10.1186/1754-6834-6-41 23514094 PMC3620520

[5] Dashtban M , Schraft H , Syed TA , Qin W . 2010. Fungal biodegradation and enzymatic modification of lignin. Int J Biochem Mol Biol 1:36–50. 21968746 PMC3180040

[6] Brock PM , Döring H , Bidartondo MI . 2009. How to know unknown fungi: the role of a herbarium. New Phytol 181:719–724. doi:10.1111/j.1469-8137.2008.02703.x 19076294

[7] Lee JS , Lim MO , Cho KY , Cho JH , Chang SY , Nam DH . 2006. Identification of medicinal mushroom species based on nuclear large subunit rDNA sequences. J Microbiol 44:29–34. 16554714

[8] Rinkes ZL , Bertrand I , Amin BAZ , Grandy AS , Wickings K , Weintraub MN . 2016. Nitrogen alters microbial enzyme dynamics but not lignin chemistry during maize decomposition. Biogeochemistry 128:171–186. doi:10.1007/s10533-016-0201-0

[9] Treseder KK . 2008. Nitrogen additions and microbial biomass: a meta-analysis of ecosystem studies. Ecol Lett 11:1111–1120. doi:10.1111/j.1461-0248.2008.01230.x 18673384

[10] Deacon LJ , Janie Pryce-Miller E , Frankland JC , Bainbridge BW , Moore PD , Robinson CH . 2006. Diversity and function of decomposer fungi from a grassland soil. Soil Biol Biochem 38:7–20. doi:10.1016/j.soilbio.2005.04.013

[11] Freedman Z , Zak DR . 2014. Atmospheric N deposition increases bacterial laccase-like multicopper oxidases: implications for organic matter decay. Appl Environ Microbiol 80:4460–4468. doi:10.1128/AEM.01224-14 24837374 PMC4068658

[12] Hobbie SE , Eddy WC , Buyarski CR , Adair EC , Ogdahl ML , Weisenhorn P . 2012. Response of decomposing litter and its microbial community to multiple forms of nitrogen enrichment. Ecol Monogr 82:389–405. doi:10.1890/11-1600.1

[13] Valdez ZP , Hockaday WC , Masiello CA , Gallagher ME , Philip Robertson G . 2017. Soil carbon and nitrogen responses to nitrogen fertilizer and harvesting rates in switchgrass cropping systems. Bioenerg Res 10:456–464. doi:10.1007/s12155-016-9810-7

[14] Moorhead DL , Lashermes G , Sinsabaugh RL , Weintraub MN . 2013. Calculating co-metabolic costs of lignin decay and their impacts on carbon use efficiency. Soil Biol and Biochem 66:17–19. doi:10.1016/j.soilbio.2013.06.016

[15] Lemaître T , Gaufichon L , Boutet-Mercey S , Christ A , Masclaux-Daubresse C . 2008. Enzymatic and metabolic diagnostic of nitrogen deficiency in Arabidopsis thaliana Wassileskija accession. Plant Cell Physiol 49:1056–1065. doi:10.1093/pcp/pcn081 18508804

[16] Fujii K , Uemura M , Hayakawa C , Funakawa S , Kosaki T . 2013. Environmental control of lignin peroxidase, manganese peroxidase, and laccase activities in forest floor layers in humid Asia. Soil Biol Biochem 57:109–115. doi:10.1016/j.soilbio.2012.07.007

[17] Kachlishvili E , Penninckx MJ , Tsiklauri N , Elisashvili V . 2006. Effect of nitrogen source on lignocellulolytic enzyme production by white-rot basidiomycetes under solid-state cultivation. World J Microbiol Biotechnol 22:391–397. doi:10.1007/s11274-005-9046-8

[18] Kumar VP , Naik C , Sridhar M . 2018. Morphological and phylogenetic identification of a hyper Laccase producing strain of Schizophyllum commune NI-07 exhibiting delignification potential. Indian J Biotechnol 17:302–315. doi:http://nopr.niscpr.res.in/handle/123456789/45094

[19] Vandana T , Kumar SA , Swaraj S , Manpal S . 2019. Purification, characterization, and bio delignification potential of lignin peroxidase from immobilized Phanerochaete chrysosporium. BioRes 14:5380–5399. doi:10.15376/biores.14.3.5380-5399

[20] Rao RG , Ravichandran A , Kandalam G , Kumar SA , Swaraj S , Sridhar M . 2019. Screening of wild basidiomycetes and evaluation of the biodegradation potential of dyes and lignin by manganese peroxidases. BioRes 14:6558–6576. doi:10.15376/biores.14.3.6558-6576

[21] Ravichandran A , Rao RG , Thammaiah V , Gopinath SM , Sridhar M . 2019 A versatile peroxidase from Lentinus squarrosulus towards enhanced delignification and in vitro digestibility of crop residues. BioRes 14:5132–5149. doi:10.15376/biores.14.3.5132-5149

[22] Kumar VP , Sridhar M , Rao RG . 2022. Biological depolymerization of lignin using laccase harvested from the autochthonous fungus Schizophyllum commune employing various production methods and its efficacy in augmenting in vitro digestibility in ruminants. Sci Rep 12:11170. doi:10.1038/s41598-022-15211-9 35778516 PMC9249777

[23] Ravichandran A , Rao RG , Gopinath SM , Sridhar M . 2021. Augmenting versatile peroxidase production from Lentinus squarrosulus and its role in enhancing ruminant feed. BioRes 16:1600–1615. doi:10.15376/biores.16.1.1600-1615

[24] G Rao R , Ravichandran A , Kandalam G , Ashish Kumar S , Swaraj S , Sridha M . 2019. Enhanced production of manganese peroxidase from Clitopilus scyphoides employing statistical optimization for application in improving crop residue digestibility by ruminants. JDVAR 8:190–203. doi:10.15406/jdvar.2019.08.00267

[25] Grąz M , Jarosz-Wilkołazka A . 2011. Oxalic acid, versatile peroxidase secretion and chelating ability of Bjerkandera fumosa in rich and limited culture conditions. World J Microbiol Biotechnol 27:1885–1891. doi:10.1007/s11274-010-0647-5 21892253 PMC3140919

[26] Lindeberg G , Holm G . 1952. Occurrence of tyrosinase and laccase in fruit bodies of mycelia of some hymenomycetes. Physiol Plant 5:100–114. doi:10.1111/j.1399-3054.1952.tb08234.x

[27] Origin pro, V2022B. 2022. Origin Lab Corporation, Northampton, MA, USA.

[28] R Studio Team . 2022. RStudio: integrated development environment for R. RStudio, PBC, Boston, MA. http://www.rstudio.co.

[29] Niku-Paavola M-L , Karhunen E , Kantelinen A , Viikari L , Lundell T , Hatakka A . 1990. The effect of culture conditions on the production of lignin modifying enzymes by the white-rot fungus Phlebia radiata. J Biotechnol 13:211–221. doi:10.1016/0168-1656(90)90106-L

[30] Fiţigău IF , Peter F , Boeriu CG . 2013. Oxidative polymerization of lignins by laccase in water-acetone mixture. Acta Biochim Pol 60:817–822. 24432339

[31] Hatakka A . 2001. Biodegradation of lignin, p 129–180. In Hofrichter M , A Steinbuchel (ed), Biopolymers 1: lignin, humic substances and coal. Wiley-VCH, Weinheim.

[32] Huang S , Huang D , Wu Q , Hou M , Tang X , Zhou J . 2020. Effect of environmental C/N ratio on activities of lignin-degrading enzymes produced by Phanerochaete chrysosporium. Pedosphere 30:285–292. doi:10.1016/S1002-0160(17)60391-6

[33] Addleman K , Dumonceaux T , Paice MG , Bourbonnais R , Archibald FS . 1995. Production and characterization of trametes versicolor mutants unable to bleach hardwood kraft pulp. Appl Environ Microbiol 61:3687–3694. doi:10.1128/aem.61.10.3687-3694.1995 16535150 PMC1388712

[34] Dawel G , Kastner M , Michels J , Poppitz W , Gunther W , Fritsche W . 1997. Structure of a laccase-mediated product of a coupling of 2,4-diamino-6-nitrotoluene to guaiacol, a model for coupling of 2,4,6-trinitrotoluene metabolites to a humic organic soil matrix. Appl Environ Microbiol 63:2560–2565. doi:10.1128/aem.63.7.2560-2565.1997 16535637 PMC1389192

[35] Kuntal K , Chauhan RS , Shavez M , Sachdeva S . 2013. Isolation of laccase producing Trichoderma sp and effect of pH and temperature on its activity. Int J Chem Environ Technol 5:2229–2235.

[36] Chowdhary P , Shukla G , Raj G , Ferreira LFR , Bharagava RN . 2019. Microbial manganese peroxidase: a ligninolytic enzyme and its ample opportunities in research. SN Appl Sci 1:45. doi:10.1007/s42452-018-0046-3

[37] Kadnikova EN , Kostić NM . 2002. Oxidation of ABTS by hydrogen peroxide catalyzed by horseradish peroxidase encapsulated into the sol-gel glass. effects of glass matrix on reactivity. Journal of Molecular Catalysis B: Enzymatic 18:39–48. doi:10.1016/S1381-1177(02)00057-7

[38] Wang G , Zhang X , Yinglan A , Duan L , Xue B , Liu T . 2021. A spatiotemporal cross-comparison framework for the accuracies of remotely sensed soil moisture products in a climate-sensitive grassland region. J Hydrol 597:126089. doi:10.1016/j.jhydrol.2021.126089

[39] Yan S , Wu G . 2017. Reorganization of gene network for degradation of polycyclic aromatic hydrocarbons (PAHs) in Pseudomonas aeruginosa PAO1 under several conditions. J Appl Genet 58:545–563. doi:10.1007/s13353-017-0402-9 28685384 PMC5655620

[40] Elisashvili V , Kachlishvili E , Penninckx M . 2008. Effect of growth substrate, method of fermentation, and nitrogen source on lignocellulose-degrading enzymes production by white-rot basidiomycetes. J Ind Microbiol Biotechnol 35:1531–1538. doi:10.1007/s10295-008-0454-2 18716810

[41] Di Lonardo DP , van der Wal A , Harkes P , de Boer W . 2020. Effect of nitrogen on fungal growth efficiency. Plant Biosyst 154:433–437. doi:10.1080/11263504.2020.1779849

[42] Lashermes G , Gainvors-Claisse A , Recous S , Bertrand I . 2016. Enzymatic strategies and carbon use efficiency of a litter-decomposing fungus grown on maize leaves, stems, and roots. Front Microbiol 7:1315. doi:10.3389/fmicb.2016.01315 27617006 PMC4999447

[43] Costa E , Teixidó N , Usall J , Atarés E , Viñas I . 2002. The effect of nitrogen and carbon sources on growth of the biocontrol agent pantoea agglomerans strain CPA-2. Lett Appl Microbiol 35:117–120. doi:10.1046/j.1472-765x.2002.01133.x 12100585

[44] Kirk TK , Farrell RL . 1987. Enzymatic combustion: the microbial degradation of lignin. Annu Rev Microbiol 41:465–505. doi:10.1146/annurev.mi.41.100187.002341 3318677

[45] Safety data sheet acc. to safe work Australia - code of practice. 2021. Azure B (C.I. 52010) for microscopy article number: 7637 version: GHS 2.0 en replaces version of 2018-07-23 version

[46] Majcherczyk A , Johannes C , Hüttermann A . 1998. Oxidation of polycyclic aromatic hydrocarbons (PAH) by laccase of Trametes versicolor. Enzyme and Microb Technol 22:335–341. doi:10.1016/S0141-0229(97)00199-3

[47] Kamitsuji H , Honda Y , Watanabe T , Kuwahara M . 2004. Production and induction of manganese peroxidase isozymes in a white-rot fungus Pleurotus ostreatus. Appl Microbiol Biotechnol 65:287–294. doi:10.1007/s00253-003-1543-9 14767623

[48] Horisawa S , Ando H , Ariga O , Sakuma Y . 2015. Direct ethanol production from cellulosic materials by consolidated biological processing using the wood rot fungus Schizophyllum commune. Bioresour Technol 197:37–41. doi:10.1016/j.biortech.2015.08.031 26318920

[49] Kumar VP , Naik C , Sridhar M . 2015. Production, purification and characterization of novel laccase produced by Schizophyllum commune NI-07 with potential for delignification of crop residues. Appl Biochem Microbiol 51:432–441. doi:10.1134/S0003683815040080

[50] Dittmer JK , Patel NJ , Dhawale SW , Dhawale SS . 1997. Production of multiple laccase isoforms by Phanerochaete chrysosporium grown under nutrient sufficiency. FEMS Microbiol Lett 149:65–70. doi:10.1111/j.1574-6968.1997.tb10309.x

[51] Chivukula M , Renganathan V . 1995. Phenolic azo dye oxidation by laccase from Pyricularia oryzae. Appl Environ Microbiol 61:4374–4377. doi:10.1128/aem.61.12.4374-4377.1995 16535191 PMC1388656

[52] Haq I , Raj A . 2018. Biodegradation of Azure-B dye by Serratia liquefaciens and its validation by phytotoxicity, genotoxicity and cytotoxicity studies. Chemosphere 196:58–68. doi:10.1016/j.chemosphere.2017.12.153 29291515

[53] Saranraj P , Sivasakthi S , Jayaprakash A . 2018. Studies on the effect of carbon and nitrogen sources for the decolorization of reactive textile dyes by bacterial isolates. World Appl Sci J 36:767–773. doi:10.5829/idosi.wasj.2018.767.773

[54] Kumaran S , Ngo ACR , Schultes FPJ , Saravanan VS , Tischler D . 2022. In vitro and in silico analysis of brilliant black degradation by actinobacteria and a Paraburkholderia sp. Genomics 114:110266. doi:10.1016/j.ygeno.2022.01.003 35031427

[55] Mancilla RA , Canessa P , Manubens A , Vicuña R . 2010. Effect of manganese on the secretion of manganese-peroxidase by the basidiomycete Ceriporiopsis subvermispora. Fungal Genet Biol 47:656–661. doi:10.1016/j.fgb.2010.04.003 20434578

[56] Kuan IC , Tien M . 1993. Stimulation of Mn peroxidase activity: a possible role for oxalate in lignin biodegradation. Proc Natl Acad Sci U S A 90:1242–1246. doi:10.1073/pnas.90.4.1242 8433984 PMC45848

[57] Hofrichter M . 2002. Review: lignin conversion by manganese peroxidase (MnP). Enzyme Microb Technol 30:454–466. doi:10.1016/S0141-0229(01)00528-2

[58] Knop D , Levinson D , Makovitzki A , Agami A , Lerer E , Mimran A , Yarden O , Hadar Y . 2016. Limits of versatility of versatile peroxidase. Appl Environ Microbiol 82:4070–4080. doi:10.1128/AEM.00743-16 27129968 PMC4959207

[59] Barber-Zucker S , Mindel V , Garcia-Ruiz E , Weinstein JJ , Alcalde M , Fleishman SJ . 2022. Stable and functionally diverse versatile peroxidases designed directly from sequences. J Am Chem Soc 144:3564–3571. doi:10.1021/jacs.1c12433 35179866 PMC8895400

[60] Milagres AMF , Arantes V , Medeiros CL , Machuca A . 2002. Production of metal chelating compounds by white and brown-rot fungi and their comparative abilities for pulp bleaching. Enz Microb Technol 30:562–565. doi:10.1016/S0141-0229(02)00015-7

[61] Singh D , Chen S . 2008. The white-rot fungus Phanerochaete chrysosporium: conditions for the production of lignin-degrading enzymes. Appl Microbiol Biotechnol 81:399–417. doi:10.1007/s00253-008-1706-9 18810426

