# Supplementary material for: Elucidating the role of media nitrogen in augmenting the production of lignin-depolymerizing enzymes by white-rot fungi
Source: Microbiol Spectr. 2023 Sep 1;11(5):e01419-23. doi: 10.1128/spectrum.01419-23 (PMC10581151; doi:10.1128/spectrum.01419-23)
Supplement: Fig. S1 — Time course plot of biomass, laccase, and peroxidase across different production methods. [file spectrum.01419-23-s0001.docx]

**Fig. S1**


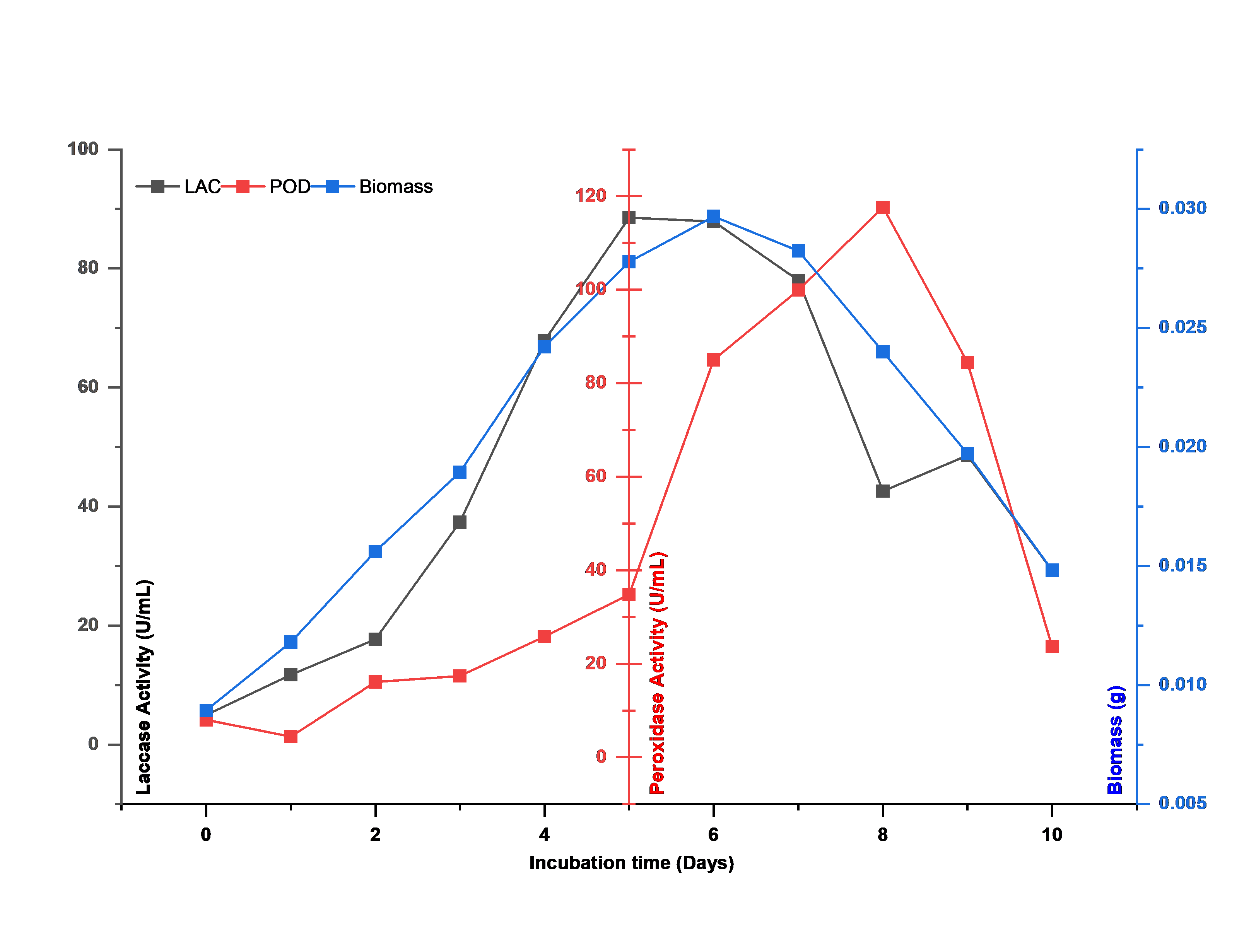


**Legend: Fig. S1**

**Time course plot of Biomass, enzymes laccase, and peroxidase (U/mL) across different production methods, in all four cultures of WRF cultures through ten days of incubation. For visual representation redundant outliers in the dataset were removed and the multi-panel/axis 3Y plot was generated to create the line and symbol plot with three different y-axis pooling average values across cultures and media for LAC (U/mL), POD (U/mL), and biomass (g). Incubation time (Days) was assigned to one X-axis****. The plot has been generated using Origin Pro 2022b. One unit (1U) of enzyme activity is defined as µmoles of product formed per minute.**
