# Supplementary material for: Elucidating the role of media nitrogen in augmenting the production of lignin-depolymerizing enzymes by white-rot fungi
Source: Microbiol Spectr. 2023 Sep 1;11(5):e01419-23. doi: 10.1128/spectrum.01419-23 (PMC10581151; doi:10.1128/spectrum.01419-23)
Supplement: Fig. S2 — Mean, median, and variance between total laccase and peroxidase across different production methods. [file spectrum.01419-23-s0002.docx]

**Fig S2**

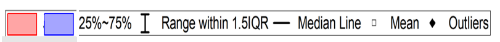


**Legend: Fig. S2**

**The Box plot shows the mean, median, and variance between total laccase and peroxidase across different production methods in all four white rot fungal isolates cultures. The square boxes represent the total activities of laccase and peroxidases in U/mL across groups. The horizontal lines crossing the boxes, below the mean, represent the median. The plots have been generated using Origin Pro 2022b. One unit (1U) of enzyme activity is defined as µmoles of product formed per minute.**
