# Supplementary material for: Elucidating the role of media nitrogen in augmenting the production of lignin-depolymerizing enzymes by white-rot fungi
Source: Microbiol Spectr. 2023 Sep 1;11(5):e01419-23. doi: 10.1128/spectrum.01419-23 (PMC10581151; doi:10.1128/spectrum.01419-23)
Supplement: Fig. S3 — Assay for LAC and POD in liquid cultures. [file spectrum.01419-23-s0003.docx]

**Fig S3**

**Assay for LAC**

**Assay for POD**


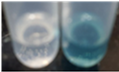


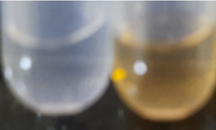


**E**

**C**

**C**

**E**

**Legend: Fig S3**

**Assay for LAC and POD in liquid cultures: C: control; E-Enzyme. One unit of LAC activity was defined as µmoles of ABTS oxidized per minute. One unit of POD activity was defined as µmoles of guaiacol oxidized per minute.**
